# Pediatric elbow arthroscopy: clinical outcomes and complications after long-term follow-up

**DOI:** 10.1186/s10195-021-00619-2

**Published:** 2021-12-20

**Authors:** Gian Mario Micheloni, Luigi Tarallo, Alberto Negri, Andrea Giorgini, Giovanni Merolla, Giuseppe Porcellini

**Affiliations:** 1 Department of Orthopaedic Surgery, Azienda Ospedaliero Universitaria Di Modena, University of Modena and Reggio Emilia, Modena, Italy; 2 Doctorate School in Clinical and Experimental Medicine, UNIMORE, Modena, Italy

**Keywords:** Elbow arthroscopy, Pediatric, Osteochondritis dissecans, Posttraumatic stiffness, Posterior impingement

## Abstract

**Background:**

Elbow arthroscopy is becoming increasingly important for the treatment of a wide range of acute and chronic elbow pathologies. Even if elbow arthroscopy is technically demanding, in the pediatric population this minimally invasive technique is preferred by many surgeons for the treatment of pathologies such as osteochondritis dissecans (OCD), posttraumatic stiffness (PTS), or elbow posterior impingement (PI). The aim of this study is to evaluate outcomes and safety of elbow arthroscopy in the pediatric and adolescent population after long-term follow-up.

**Materials and methods:**

In this retrospective study, 26 patients younger than 18 years old undergoing elbow arthroscopy were evaluated. All surgeries were performed by a single senior surgeon. Patients were divided into three subgroups based on preoperative diagnosis: OCD, PTS, and PI. After at least 60 months follow-up, several outcome measures, including range of motion (ROM), Mayo Elbow Performance Score (MEPS), and visual analog scale (VAS) were evaluated in relation to preoperative values. The level of patient satisfaction on a five-level Likert scale, any limitation or change in sport activity, and the onset of any possible complications were also evaluated.

**Results:**

In the study population, we found an improvement in ROM (flexion of 14.4 ± 13.6°, extension of 19.5 ± 13.9°, pronation of 5.8 ± 5.7°, and supination of 8.5 ± 11.6°) and in validated outcome measures (MEPS of 21.0 ± 13.5 points and VAS of 3.8 ± 2.2 points). The satisfaction rate was 4.5, with no dissatisfaction. Eighty-seven percent of patients fully recovered their performance levels, 9% changed sport, and 4% were unable to return to sport. We identified one major and one minor complication, with an overall complication rate of 7.7%. No neurovascular injuries were detected.

**Conclusions:**

Elbow arthroscopy in a pediatric population can be considered an effective and safe procedure for selected pathologies when performed by an experienced surgeon. At long-term follow-up, we reported excellent clinical outcomes (both objective and subjective), with a relatively low complication rate without permanent injuries.

**Level of evidence:**

Level IV—case series.

## Introduction

Elbow arthroscopy has only recently developed due to its technical difficulty, the complex joint anatomy, and the proximity of surrounding neurovascular structures (such as the medial, ulnar, and radial nerves, as well as the brachial artery) [1]. Elbow arthroscopy is even more demanding in pediatric and adolescent patients, where joint space is tighter and there is greater concern that any complications may permanently affect growth [2, 3].

Nevertheless, due to significant advances in arthroscopic technology and equipment, coupled with the expansion of knowledge about joint anatomy and increased experience of orthopedic surgeons, elbow arthroscopy has acquired an increasingly important role in recent years. Given the less invasive nature of arthroscopic surgical techniques, with reduced postoperative pain, lower rate of surgical wound complications, reduced infection risk, and easier postoperative rehabilitation program, surgeons prefer arthroscopic treatment for a wide range of acute and chronic elbow pathologies [2, 4–7].

Although most of the available literature concerns elbow arthroscopy in adults, this mini-invasive technique has also emerged in the pediatric population as a safe and effective treatment for several pathologies [3, 8, 9]. Osteochondritis dissecans (OCD) is one of the pediatric elbow diseases for which arthroscopic treatment is most frequently indicated [10–12].

Posttraumatic elbow stiffness (PTS) is quite a common elbow injury complication in the pediatric population. Elbow fractures are common in children, with an incidence rate of 5–10% of all fractures [13]. The complex pediatric anatomy makes radiograph interpretation demanding, thus leading to an incorrect assessment of the lesion’s complexity and, therefore, to inadequate treatment. Predisposing factors for stiffness are length of immobilization and the severity of injury, with an increased risk of intraarticular fracture malunion, degenerative changes, heterotopic ossification, and soft tissue contracture [14]. Indications for arthroscopic arthrolysis are failure of 6 months conservative treatment or stiffness with impairment of daily activities or sport performances due to severe ROM limitation [15, 16].

Another pathology that can benefit from arthroscopic treatment is elbow posterior impingement (PI), characterized by disabling pain and limitation of ROM, especially in extension. Elbow weight-bearing sports may expose the elbow to repeated hyperextension trauma [17, 18] and valgus extension overload syndrome [19]. These excessive forces can lead to elbow degenerative changes such as osteophytes, loose bodies, and capsular stiffness, not only in the adult population but also among adolescents [20].

The aim of this study is to evaluate the safety of elbow arthroscopy in the pediatric and adolescent population and outcomes after long-term follow-up. Our hypothesis is that most pediatric patients achieve good to excellent clinical outcomes and a complete return to sport activities after elbow arthroscopy with a low complication rate.

## Materials and methods

This is a retrospective study on patients aged 18 years or younger, undergoing elbow arthroscopy in our institute between 2010 and 2015. Inclusion criteria were preoperative diagnosis of posttraumatic elbow stiffness (PTS), osteochondritis dissecans (OCD), or posterior impingement (PI), with no other disabling pathology, no polytrauma patients, or failure of conservative treatment (at least 6 months). All procedures were performed by a single expert surgeon (G.P., with more than 30 elbow arthroscopic procedures per year). Twenty-nine patients were recruited, of which, three were lost at follow-up (dropout rate 10%).

Preoperative patient records were reviewed to collect information about the patient’s age at the time of surgery, sex, laterality and dominant hand, sport practiced, first diagnosis, comorbidities, clinical evaluation [including Mayo Elbow Performance score (MEPS)] and pain on visual analog scale (VAS) (Table 1). Radiographic images [anteroposterior (AP) and lateral (LL) view] and computed tomography (CT) scans were evaluated.

**Table 1:** Demographic and anamnestic data of the study population

Case	Sex	Age (years)	Diagnosis	Side (D/n)	Sport
1	M	12	OCD	L (n)	Soccer
2	F	17	OCD	R (D)	Basketball
3	M	18	OCD	L (D)	Tennis
4	M	16	OCD	L (n)	Rugby
5	M	17	OCD	R (D)	Swimming
6	M	16	OCD	R (D)	Basketball
7	F	14	OCD	R (D)	Artistic gymnastics
8	M	14	OCD	L (D)	Soccer
9	M	18	OCD	L (D)	Baseball
10	M	13	OCD	L (D)	Soccer
11	F	12	PTS	L (n)	Swimming
12	M	18	PTS	L (D)	Swimming
13	M	15	PTS	R (n)	Tennis
14	F	13	PTS	R (D)	Volleyball
15	F	12	PTS	L (n)	Athletics
16	M	17	PTS	L (n)	Athletics
17	M	8	PTS	L (n)	No sport
18	F	14	PTS	L (D)	Volleyball
19	F	15	PTS	L (D)	No sport
20	M	18	PTS	L (n)	Karate
21	F	15	PTS	R (D)	Boxing
22	F	17	PTS	L (n)	Athletics
23	F	14	PI	R (D)	No sport
24	M	18	PI	R (D)	Baseball
25	F	15	PI	L (n)	Volleyball
26	M	15	PI	R (D)	Baseball

Characteristics of the study population with case identification number, sex, age at the time of surgery (years), preoperative diagnosis group (*OCD* osteochondritis dissecans, *PTS* posttraumatic elbow stiffness, *PI* posterior impingement), side of the affected elbow (right/left, *D* dominant side, *n* not dominant), preoperative sport activity practiced

Mean age at the time of surgery was 15 years old (SD: 2.5; range: 8–18 years) with 15 males (58%) and 11 females (42%). Sixteen patients (62%) were treated for pathology of the left elbow, 10 for the right (38%), and 62% of the procedures were conducted on the dominant side.

Patients were divided into three groups based on preoperative diagnosis: 12 (46%) underwent elbow arthroscopy due to PTS, 10 (39%) for OCD, and 4 (15%) complained of loss of elbow extension for PI. In the PTS group, arthrofibrosis was secondary to a recognized past elbow fracture in nine patients (75%), while three patients (25%) reported only traumatic events without clear lesions. The fractures identified in anamnesis were: three radial head fractures (33%), three lateral condyle fractures (33%), two supracondylar humeral fractures (22%), and one intraarticular distal humeral fracture (11%). Only patients with OCD types I and II (Clanton and DeLee classification) were included in the study. Patients with grades III and IV OCD were treated with open procedures and therefore excluded from the cohort. Twenty-three patients (88%) practiced high-level sports (in junior categories), while three patients (12%) practiced noncompetitive sports (Table 1).

The patients were examined after a follow-up of at least 60 months (mean 66.8 months, SD: 5.3, range: 60–78 months) by one independent surgeon. The examination included measurement of the elbow range of motion (ROM) with a goniometer and the validated outcome measures MEPS and VAS, comparing them with the preoperative ones taken at the time of surgery. Patients were also questioned about the time required to sports return and any limitation or change in sport activity after surgery. The postoperative satisfaction was recorded with a five-level Likert scale. Finally, standard anterior–posterior and lateral radiographs were taken for all patients to confirm the postoperative result and to identify any pathological findings. Postoperative complications were evaluated. These were divided in two categories: major complications when further surgical intervention was required and minor complications when conservative treatment was adopted.

Statistical analysis was performed using STATA® software version 14 (StataCorp. 2015. Stata Statistical Software: Release 14. College Station, TX: StataCorp LP.). Continuous variables were presented as the number of patients (N), mean, standard deviation (SD), minimum (min), and maximum (max), and compared between subgroups using unpaired Student’s *t*-test for two groups, or ANOVA for more than two groups;. Categorical variables were presented as frequency [*N*, percentage (%)] and compared using Pearson’s chi‐squared test. Margins were statistically calculated from predictions of a previously fitted model at fixed values of some covariates, and averaging or otherwise integrating the remaining covariates. Capabilities include estimated marginal means, least-square means, average and conditional marginal and partial effects (which may be reported as derivatives or as elasticities), average and conditional adjusted predictions, and predictive margins. A *P* < 0.05 was considered statistically significant.

### Surgical technique and rehabilitation

All surgeries were performed under intravenous anesthesia and preoperative antibiotics were administered. Patients were placed in a lateral decubitus position. The operative arm was supported with an arm holder to allow complete arthroscopic examination of the elbow joint and without mechanical impediments to perform ROM examination and stability tests during the procedure. A tourniquet was placed high on the upper arm to control bleeding until the end of surgery.

**Fig. 1:**
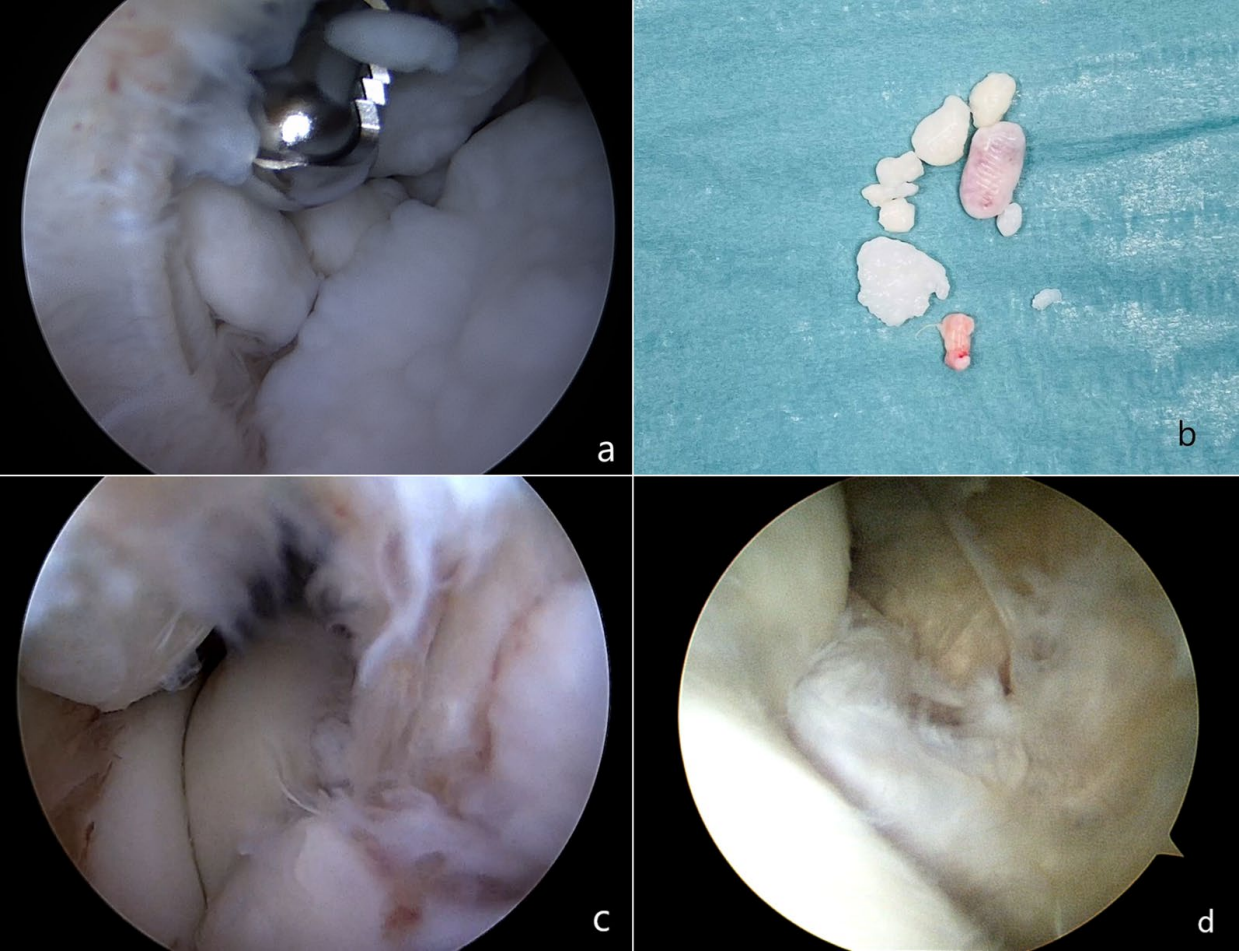
Intraoperative arthroscopic pathological findings. Intraoperative arthroscopic pathological findings. **a** loose bodies identified during arthroscopic exploration of the joint cavity. **b** Some loose bodies removed from the anterior and the posterior compartments during the procedure. **c**, **d** extensive arthrofibrosis involving the radiocapitellar joint and the medial joint compartment (humeral trochlea)

A standard 4-mm arthroscope with a 30° angled optic lens was used with a pressure-controlled pump. Bony landmarks were mapped, including radiocapitellar joint, ulnar nerve, olecranon, medial and lateral epicondyle, and the joint space was distended with an intraarticular injection of 20 ml saline solution through the soft spot. Two main anterior (proximal anterolateral, proximal anteromedial) and posterior (posterolateral, direct posterior) portals were used and accessory portals (soft spot, anteromedial, and anterolateral) were used if necessary. In all procedures, both the anterior and the posterior compartments were examined (Fig. 1a–d).

In the PTS group, joint arthrolysis was performed associated to synovectomy in four cases (33%), with the removal of loose bodies in another four cases (33%), and the removal of osteophytes in two cases (17%). In the OCD group (types I and II according to Clanton and DeLee classification), cartilage debridement was performed, removing flaps and other irregularities. Several procedures were associated with these arthroscopies: six synovectomy (67%), five microfractures (56%), four removals of loose bodies (44%), and three arthrolysis (33%). For the PI group, elbow posterior compartment debridement was performed, with two cases of osteophytes removal (75%). No mini-open procedures for ulnar nerve debridement were performed.

After all surgical steps were performed, the final ROM under anesthesia was examined by the surgeon; varus and valgus stress tests were performed to confirm elbow stability. At the end of the procedure, intraarticular drains were placed to prevent hemarthrosis, and all portals were closed using absorbable sutures. The drains were removed generally on the first postoperative day.

Postoperatively, the elbow was placed in maximum extension in a posterior long-arm splint. The splint was removed on the second day to allow rehabilitation. The beginning of physical therapy and the rehabilitation program varied from patient to patient and was related to the diagnosis [21]. All patients begun passive motion on the next day after the surgery if pain and swelling were tolerable. Continuous passive motion was administered to all patients for 2 weeks. Water-assisted exercises were allowed from 21 days postoperative to 45 days. Strengthening with weight and elastic resistance were allowed after 45 days. Athletes restarted swimming after 2 months and artistic gymnastic after 6 months.

## Results

Overall clinical outcomes are reported in Table 2. In the PTS group (Table 3), the mean postoperative flexion increased by 21.7° (SD: 13.0, *P* = 0.0001) compared with preoperatively; this improvement was statistically significant compared with the other groups. The mean extension increased by 20.4° (SD: 15.1, *P* = 0.0007), the mean pronation by 6.7° (SD: 6.5, *P*  = 0.0046), and the mean supination by 12.1° (SD: 21.8, *P* = 0.0193). The preoperative pain reported on VAS score was 3 (SD: 2.1) points. At final follow-up, five patients (42%) reported mild pain during most demanding activities, while seven patients (58%) reported no pain, with an overall postoperative VAS score of 0.6 points (SD: 0.8, *P* = 0.0007). The validated MEPS score showed an improvement from 76.3 (SD: 19.3) points to 93.8 (SD: 8.6, *P* = 0.0051) points postoperatively.

**Table 2:** Total study population clinical outcomes (follow-up 60 months)

	Preoperative (SD, range)	Postoperative (SD, range)	Variation (SD)	*P*-value
Flexion	122.1° (SD: 19.4°, 80°/145°)	136.5° (SD: 9.3°, 120°/145°)	+14.4° (SD: 13.6°, +0°/+50°)	< 0.0001
Extension	22.0° (SD: 19.2°, 60°/−10°)	2.5° (SD: 9.9°, 20°/−10°)	+ 19.5° (SD: 13.9°, +0°/+50°)	< 0.0001
Pronation	81.9° (SD: 7.9°, 60°/90°)	87.7° (SD: 4.2°, 80°/90°)	+5.8° (SD: 5.7°, +0°/+20°)	< 0.0001
Supination	78.5° (SD: 15.4°, 30°/90°)	87.0° (SD: 6.7°, 60°/90°)	+ 8.5° (SD: 11.6°, +0°/+ 40°)	0.0007
VAS score	4.5 (SD: 2.5, 8/0)	0.7 (SD: 0.9, 3/0)	−3.8 (SD: 2.2, −0/−7)	< 0.0001
MEPS score	73.8 (SD: 14.7, 25/95)	94.8 (SD: 7.6, 80/100)	+ 21.0 (SD: 13.5, +15/+55)	< 0.0001

Overall clinical outcomes of the study population. The first four lines report the basic movements of the elbow: flexion, extension, pronation, and supination (in degrees). The last two lines show the VAS score and the MEPS score in points. For each element, the preoperative and postoperative means (with *SD* standard deviation and range with minimum / maximum value obtained), the variation, and the *P*-value are reported

**Table 3:** PTS group clinical outcomes (follow-up 60 months)

	Preoperative (SD, range)	Postoperative (SD, range)	Variation (SD)	*P*-value
Flexion	109.2° (SD: 19.3°, 80°/140°)	130.8° (SD: 10.0°, 120°/145°)	+21.7° (SD: 13.0°, +0°/+50°)	0.0001
Extension	24.2° (SD: 23.1°, 60°/−10°)	3.8° (SD: 12.3°, 20°/−10°)	+20.4° (SD: 15.1°, +0°/+50°)	0.0007
Pronation	79.2° (SD: 10.0°, 60°/90°)	85.8° (SD: 5.1°, 80°/90°)	+6.7° (SD: 6.5°, +0°/+20°)	0.0046
Supination	72.9° (SD: 20.7°, 30°/90°)	85.0° (SD: 9.0°, 80°/90°)	+12.1° (SD: 21.8°, +0°/+40°)	0.0193
VAS score	3.0 (SD: 2.1, 7/0)	0.6 (SD: 0.8, 2/0)	−2.4 (SD: 1.8, −0/−5)	0.0007
MEPS score	76.3 (SD: 19.3, 25/95)	93.8 (SD: 8.6, 80/100)	+ 17.5 (SD: 17.4, +15/+55)	0.0051

Overall clinical results of the PTS group patients. For each measured outcome (flexion, extension, pronation, supination, VAS score, and MEPS score) the preoperative and postoperative means (with *SD* standard deviation and range with minimum / maximum value obtained), the variation, and the *P*-value are reported

In OCD patients, ROM increased, as indicated in Table 4: the mean flexion increased by 11.5° (SD: 12.3°, *P* = 0.0158), the mean extension by 13.3° (SD: 11.5°, *P* = 0.0051), the mean pronation by 6° (SD: 5.2°, *P* = 0.0051), and the mean supination by 6.5° (SD: 7.5°, *P* = 0.0224). The pain relief reported by the patients in this group was statistically significant compared with the other groups: the overall VAS score improved from 6.4 points (SD: 1.4) preoperatively to 0.8 points (SD: 0.8, *P*: < 0.0001) after surgery. The MEPS score increased from 70 points (SD: 10.0) preoperative to 94 points postoperative (SD: 7.7, *P*: < 0.0001).

**Table 4:** OCD group clinical outcomes (follow-up 60 months)

	Preoperative (SD, range)	Postoperative (SD, range)	Variation (SD)	*P*-value
Flexion	128.5° (SD: 11.3°, 110°/145°)	140.0° (SD: 6.2°, 130°/145°)	+11.5° (SD: 12.3°, +0°/+35°)	0.0158
Extension	13.3° (SD: 13.6°, 30°/−5°)	0.0° (SD: 8.8°, 10°/−10°)	+13.3° (SD: 11.5°, +0°/+35°)	0.0051
Pronation	83.0° (SD: 4.8°, 80°/90°)	89.0° (SD: 3.2°, 80°/90°)	+6.0° (SD: 5.2°, +0°/+10°)	0.0051
Supination	81.5° (SD: 8.2°, 70°/90°)	88.0° (SD: 4.2°, 80°/90°)	+6.5° (SD: 7.5°, +0°/+20°)	0.0224
VAS score	6.4 (SD: 1.4, 8/4)	0.8 (SD: 0.8, 2/0)	−5.6 (SD: 1.5, −3/− 7)	< 0.0001
MEPS score	70.0 (SD: 10.0, 55/85)	94.0 (SD: 7.7, 85/100)	+ 24.0 (SD: 9.7, +5/+35)	< 0.0001

Overall clinical results of the OCD group patients. For each measured outcome (flexion, extension, pronation, supination, VAS score, and MEPS score) the preoperative and postoperative means (with *SD* standard deviation and range with minimum / maximum value obtained), the variation, and the *P*-value are reported

For PI group, the mean extension showed an improvement of 32.5° (SD: 8.7, *P* = 0.0049), while the mean pronation and supination both increased by 2.5° (SD: 5.0, *P* = 0.3910 each), as reported in Table 5. Postoperatively, flexion showed no changes from preoperative values. The mean VAS score increased by 0.8 points (SD: 1.5); the preoperative mean VAS score was 4.3 points (SD: 2.6, *P* = 0.0354). One patient in this group reported mild pain after surgery. The postoperative MEPS showed an improvement of 23.8 points (SD: 10.3, *P* = 0.0192) after surgery compared with the preoperative score.

**Table 5:** PI group clinical outcomes (follow-up 60 months)

	Preoperative (SD, range)	Postoperative (SD, range)	Variation (SD)	*P*-value
Flexion	145.0° (SD: 0.0°)	145.0° (SD: 0.0°)	0.0	–
Extension	37.5° (SD: 9.6°, 50°/30°)	5.0° (SD: 4.1°, 10°/0°)	+32.5° (SD: 8.7°, +25°/+45°)	0.0049
Pronation	87.5° (SD: 5.0°, 80°/90°)	90.0° (SD: 0.0°)	+2.5° (SD: 5.0°, +0°/+10°)	0.3910
Supination	87.5° (SD: 5.0°, 80°/90°)	90.0° (SD: 0.0°)	+2.5° (SD: 5.0°, +0°/+10°)	0.3910
VAS score	4.3 (SD: 2.6, 7/2)	0.8 (SD: 1.5, 3/0)	−3.5 (SD: 1.9, −2/−6)	0.0354
MEPS score	76.3 (SD: 10.3, 65/85)	100.0 (SD: 0.0)	+23.8 (SD: 10.3, +15/+35)	0.0192

Overall clinical results of the PI group patients. For each measured outcome (flexion, extension, pronation, supination, VAS score and MEPS score) the preoperative and postoperative means (with *SD* standard deviation, and range with minimum/maximum value obtained), the variation, and the *P*-value are reported

**Fig. 2:**
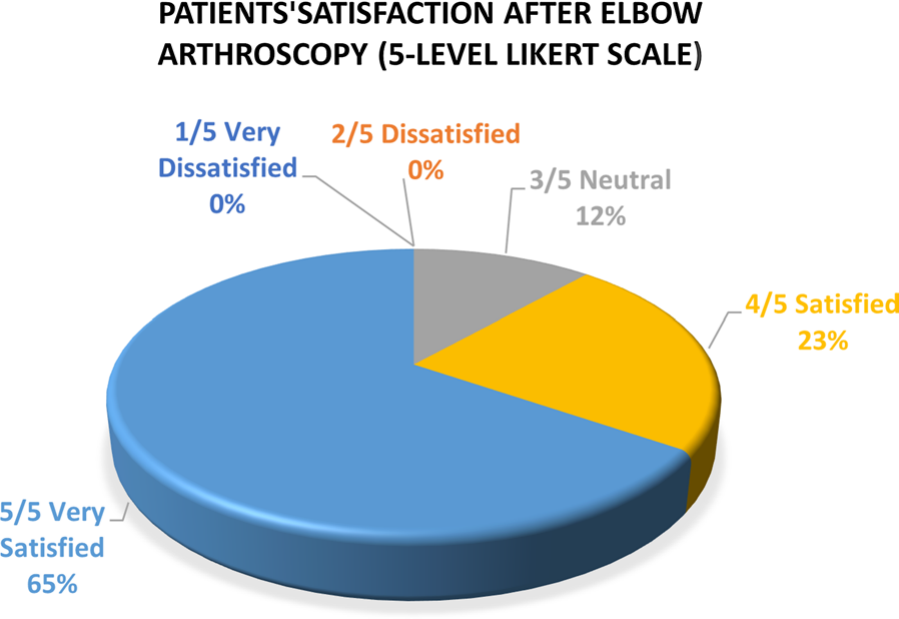
Patient satisfaction after elbow arthroscopy (five-level Likert scale). Reported patient satisfaction after elbow arthroscopy using five-level Likert scale (follow-up 60 months). No one was dissatisfied with the procedure, three patients were neutral, six were satisfied, and 17 were very satisfied after elbow arthroscopy

Satisfaction after surgery is shown in Fig. 2. Using a five-level Likert scale, the mean satisfaction rate was 4.6 (SD: 0.7). Twenty-three patients (88%) reported being “satisfied” or “very satisfied", only three (12%) reported feeling neutral (one patient in each group, PTS, OCD, and PI), and none expressed dissatisfaction after elbow arthroscopy.

**Fig. 3:**
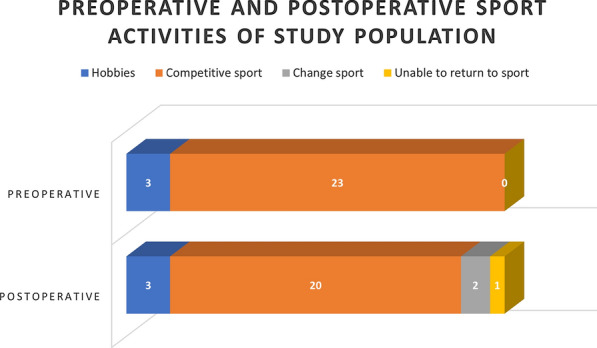
Preoperative and postoperative sport activities of the study population. Reported sport activities before and after elbow arthroscopy (follow-up 60 months). Preoperatively, 23 patients practiced several kinds of sports as presented in Table 1 and only three patients practiced noncompetitive sport activities (hobbies). Postoperatively, 20 patients involved in competitive sports continued their activities, two patients changed their disciplines, and one was unable to return to sport. The three patients who practiced noncompetitive sports continued their activities

Twenty out of twenty-three patients (87%) that practiced competitive sports were able to full recover their performance levels in their disciplines after elbow arthroscopy, as shown in Fig. 3. Patients in the OCD group returned to playing sport in about 5 months, while patients in the PTS and PI groups returned in about 2 months. The time to return to sport is related to the type of sports discipline: swimmers need less time than gymnasts or baseball players. Two patients (9%) changed their sport, while one patient (4%) was unable to return to sport (baseball) after elbow arthroscopy due to the onset of a complication described below. The three patients who practiced noncompetitive sports before surgery continued their hobbies without any limitation.

In this study, we identified one major (3.8%) and one minor (3.8%) complication, with an overall complication rate of 7.7%. The major complication occurred in a patient in the PI group, where we detected triceps insertional tendinopathy during the rehabilitation protocol. We identified the minor complication through the postoperative X-ray examination in a girl from the PTS group, 3 years after surgery some heterotopic ossifications were detected around both lateral and medial condyles. This is the only pathological finding detected by postoperative X-ray examination of our study population.

## Discussion

Elbow arthroscopy is a procedure of increasing interest for the treatment of a wide range of acute and chronic elbow pathologies. Despite the wide use of this technique in adults, literature about arthroscopic management of elbow pathologies in pediatric patients is still limited. In recent years, some authors, such as Micheli et al. [9], Vavken et al. [8], and Andelman et al. [3] have shown the main indications and results of elbow arthroscopy in the pediatric population.

In this study, we performed elbow arthroscopy for three main diagnoses: posttraumatic elbow stiffness, osteochondritis dissecans, and posterior impingement.

**Fig. 4:**
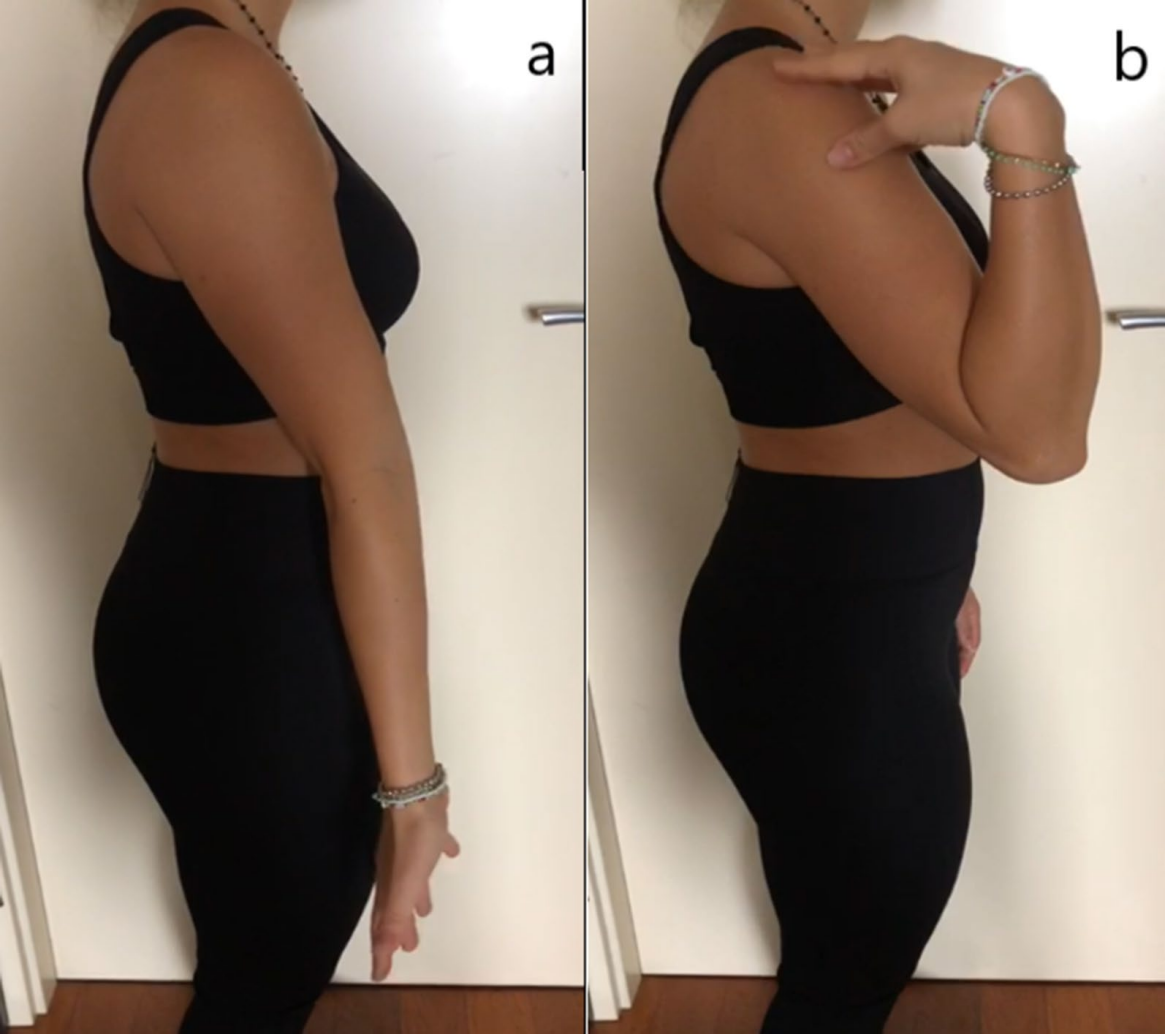
Full elbow range of motion after arthroscopy. Postoperative ROM improvement achieved after arthroscopy by one of our young athletes, with complete arc of flexion (**b**) and extension (**a**)

Our PTS group was made up of children who suffered significant elbow ROM limitation following conservative or surgical treatment for past elbow fracture (75% of cases) or other traumatic injuries: after arthroscopic arthrolysis we found an important ROM improvement for these patient at 60 months follow-up, as expected. These improvement are mainly in flexion, but extension, supination, and pronation also improved significantly. All our patients exceeded the limit of functional arc of motion identified by Morrey et al. [22] and they managed to fulfill all daily activities without any limitation (Fig. 4a, b). In this group, pain limited the ROM preoperatively. Arthroscopic arthrolysis was also effective in pain relief: patients gave an overall VAS score of 0.6 (SD: 0.8, *P* = 0.0007) points and 93.8 (SD: 8.6, *P* = 0.0051) points on MEPS, achieving excellent postoperative results. In the literature, we find similar outcomes [3, 9, 23, 24] which confirm our findings about the use of arthroscopic arthrolysis for posttraumatic elbow stiffness in reducing pain and increasing ROM.

Elbow arthroscopy in OCD treatment is considered the gold standard technique in the pediatric population, with excellent clinical results reported in the literature [3, 23, 25–27]. We performed arthroscopic debridement associated with microfractures in 56% of the cases, obtaining satisfactory long-term results. Several patients in the OCD group had a significant limitation in both sport and daily activities secondary to pain rather than capsular stiffness. In these patients, we observed the greatest VAS score improvement, from 6.4 points (SD: 1.4) preoperatively to 0.8 points (SD: 0.8, *P*: < 0.0001) after arthroscopy. As a result of reduced pain, the MEPS score and postoperative ROM also showed a significant improvement that contributed to patient satisfaction, albeit lower than the other groups.

In the PI group, we included patients with osteophytes, olecranon spur, or loose bodies in the posterior compartment that limited the elbow extension. As reported in the literature, this study confirmed a higher incidence in those patients involved sports with overloading of the upper limbs (baseball and volleyball) [17–19]. In the PI group, the greatest increase in elbow extension was reported [from 37.5° (SD: 9.6) to 5.0° (SD: 4.1); *P* = 0.0049], achieving a functional final ROM sufficient to fulfill all the activities of daily living. Interestingly, no patient reported any pain, and postoperative MEPS score was 100 points for all patients.

To make a complete evaluation of the clinical results, it is important not only to evaluate objective data but also consider the patient’s opinion regarding their expectations. For this reason, we decided to investigate patient postoperative satisfaction, finding an average increase of 4.5 points (SD: 0.7) with no dissatisfaction, which confirms the effectiveness of the procedure.

Return to sport was one of the main goals of our patients who suffered from elbow disease. After surgery on a pediatric patient, the full recovery of sport performance is a fundamental element for both the psychic and physical development of the child, and for these reason, we believe this should be considered an important outcome measure. Eighty-seven percent of our patients were able to return to their previous level of sport, continuing their athletic careers without problems. Similar results are presented in pediatric literature, in which most studies reported a high rate of sports performance after elbow arthroscopic surgery [9, 27, 28]. Two of our patients (9%) changed their sport activity after arthroscopy, one from the PTS group and the other from the OCD group. These patients reported a feeling of fear practicing their old sport (volleyball and rugby) rather than a real impossibility related to elbow functionality. For this reason, they changed the type of sport achieving good–excellent results in the new discipline anyway. Byrd et al. [28] reported some similar cases: sometimes young nonprofessional athletes preferred to change sport and start a new career, avoiding the risk of further elbow injuries. In conclusion, in our study, only one patient (4%) was unable to return to their previous sport (baseball) due to the onset of a major complication. These positive results are further evidence of the effectiveness of elbow arthroscopy in the pediatric population. However, we must consider that in our study population, there were only a few professional athletes in disciplines that overload the upper limbs, and this may have positively influenced the results.

In the available literature, the overall complication rate for elbow arthroscopy ranged from 6% to 14% [1, 2, 29–32] in large series studies in adults. Although these complications were mostly self-resolving, without requiring further surgical procedures, this complication rate is still higher than other joint arthroscopies such as knee, shoulder, or hip. The first studies on pediatric elbow arthroscopy reported a lower complication rate compared with adults: Micheli et al. [9] showed no postoperative complications (0%) while Vavken et al. [8] found a minor complication rate of 8%. However, Andelman et al. [3] reported a complication rate of 17.2%, although this is most likely the result of the high number of complex procedures performed in their study.

We reported a complication rate of 7.7% after long-term follow-up, with a major complication (triceps insertional tendinopathy) that required further open surgery and a minor complication (heterotopic ossifications) treated conservatively.

The major complication was identified in a patient undergoing arthroscopic posterior compartment debridement, removal of osteophytes, and removal of loose bodies for elbow posterior impingement. During the rehabilitation protocol, this patient showed poor results with persistent pain and functional limitation due to triceps insertional tendinopathy highlighted by ultrasound examination. This was subsequently treated with an open debridement of damaged tissues and tendon repair performed a few months after the first procedures. After this second procedure, the patient reached a good functional outcome, with 85 points on MEPS score, returning to the previously level of baseball.

**Fig. 5:**
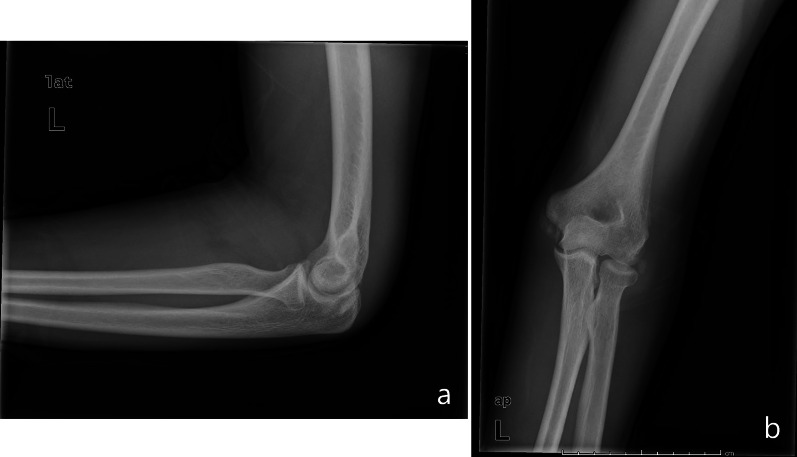
Heterotopic ossifications on X-ray examination. X-ray examination with heterotopic ossifications findings around both lateral and medial condyles (**a**, **b**)

Heterotopic ossifications appear to be a less common finding after elbow arthroscopy than after open procedures, but Gofton et al. [33] predicted an increased incidence due to the expansion of the arthroscopy for more complicated procedures than in the past. In the literature, the reported incidence rate ranges from 0.4% to 2.5% [2, 31, 34] in large adult population studies, with a high percentage of cases requiring reoperation. To our knowledge, only a study by Sodha et al. [35] identified heterotopic ossifications in a young throwing athlete. In our study, we reported a single case of heterotopic ossification in a girl treated with arthroscopic arthrolysis, removal of loose bodies, and osteophytes for posttraumatic stiffness after lateral condyle fractures (Fig. 5a, b). This patient reported excellent outcome measures and no significant ROM limitation. For this reason, a reoperation was not necessary at the final follow-up, unlike in other studies. More studies are needed to determine the increased risk and to identify the appropriate prophylaxis for heterotopic ossifications after elbow arthroscopy in the pediatric population.

We did not detect any other pathological finding with the postoperative X-ray, especially any alteration of the skeletal maturation (malalignments, growth defects), demonstrating the reduced invasiveness and safety profile of elbow arthroscopy.

A significant result of this study was the absence of neurovascular injuries despite the proximity of the arthroscopic portals to these structures. These are the most frequent complications reported in literature after elbow arthroscopy [1, 2, 30, 31]. To avoid risks, exact anatomical knowledge of the complex elbow anatomy and the experience of the surgeon are essential, especially in the pediatric population [29]. Despite the growing practice of elbow arthroscopy, there is a paucity of literature about the surgical learning curve. Only Keyt et al. [36] identified a minimum threshold of 230 elbow arthroscopies to achieve expert level performance. Generally, pediatric elbow arthroscopy is performed only by experienced surgeons to avoid the risk of permanent damage in this particular population. Probably this is a factor that justifies the low complication rate reported in our study, as in in other investigations on the pediatric population [8, 9]. Surgical technique has several constantly developing features, including patient decubitus position (lateral, supine, prone), number of portals (four principals and some accessories portals), procedure stages (anterior or posterior compartment first), and device features. From the analysis of the available literature, it is not possible to identify a gold standard [3, 8, 23, 37]. We believe that, given the difficulty and the experience required to perform these procedures, surgeons who perform a pediatric elbow arthroscopy should choose the surgical technique in which they are most experienced, to minimize the risks.

Our study has some limitations. First of all, this is a retrospective study with no control group. The small sample size is mainly due to the rarity of this pathology. Another important limitation is the heterogeneity of indications for arthroscopic procedures that limits the number of patients for each group and, thus, weakens the statistical significance. The long-term follow-up time is the strength of this work, providing a longer prospective to observe the development of outcomes and complications.

## Conclusion

Elbow arthroscopy performed by an expert surgeon in the pediatric and adolescent population is a safe procedure, with a low complication rate and a high rate of patients satisfaction at long-term follow-up. Another important finding is the high rate of return to sports at previous levels.

## Data Availability

The datasets used and/or analyzed during the current study are available from the corresponding author on reasonable request.

## Abbreviations

OCD: Osteochondritis dissecans
PTS: Posttraumatic elbow stiffness
ROM: Range of motion
PI: Posterior impingement
MEPS: Mayo Elbow Performance Score
VAS: Visual analog scale
SD: Standard deviation

## References

[1] O'Driscoll SW, Morrey BF (1992). Arthroscopy of the elbow. Diagnostic and therapeutic benefits and hazards. J Bone Joint Surg.

[2] Yeoh KM, King GJ, Faber KJ, Glazebrook MA, Athwal GS (2012). Evidence-based indications for elbow arthroscopy. Arthroscopy.

[3] Byram IR, Kim HM, Levine WN, Ahmad CS (2013). Elbow arthroscopic surgery update for sports medicine conditions. Am J Sports Med.

[4] Dodson CC, Nho SJ, Williams RJ, Altchek DW (2008). Elbow arthroscopy. J Am Acad Orthop Surg.

[5] Kelly EW, Morrey BF, O'Driscoll SW (2001). Complications of elbow arthroscopy. J Bone Joint Surg.

[6] Tarallo L, Mugnai R, Fiacchi F, Adani R, Zambianchi F, Catani F (2015). Pediatric medial epicondyle fractures with intra-articular elbow incarceration. J Orthopaedics Traumatol.

[7] Vavken P, Müller AM, Camathias C (2016). First 50 pediatric and adolescent elbow arthroscopies: analysis of indications and complications. J Pediatr Orthop.

[8] Andelman SM, Meier KM, Walsh AL, Kim JH, Hausman MR (2017). Pediatric elbow arthroscopy: indications and safety. J Shoulder Elbow Surg.

[9] Micheli LJ, Luke AC, Mintzer CM, Waters PM (2001). Elbow arthroscopy in the pediatric and adolescent population. Arthroscopy.

[10] Logli AL, Bernard CD, O'Driscoll SW, Sanchez-Sotelo J, Morrey ME, Krych AJ, Camp CL (2019). Osteochondritis dissecans lesions of the capitellum in overhead athletes: a review of current evidence and proposed treatment algorithm. Curr Rev Musculoskelet Med.

[11] Churchill RW, Munoz J, Ahmad CS (2016). Osteochondritis dissecans of the elbow. Curr Rev Musculoskelet Med.

[12] Baumgarten TE, Andrews JR, Satterwhite YE (1998). The arthroscopic classification and treatment of osteochondritis dissecans of the capitellum. Am J Sports Med.

[13] Herring JA, Ho C (2002). Upper extremity injuries. Tachdjian’s Pediatr Orthopaedics.

[14] Hyatt BT, Schmitz MR, Rush JK (2016). Complications of Pediatric Elbow Fractures. Orthop Clin North Am.

[15] Myden C, Hildebrand K (2011). Elbow joint contracture after traumatic injury. J Shoulder Elbow Surg.

[16] Bruno RJ, Lee ML, Strauch RJ, Rosenwasser MP (2002). Posttraumatic elbow stiffness: evaluation and management. J Am Acad Orthop Surg.

[17] Ahmad CS, Conway JE (2011). Elbow arthroscopy: valgus extension overload. Instr Course Lect.

[18] Moskal MJ, Savoie FH, Field LD (1999). Arthroscopic treatment of posterior elbow impingement. Instr Course Lect.

[19] O'Holleran JD, Altchek DW (2006). The thrower's elbow: arthroscopic treatment of valgus extension overload syndrome. HSS J.

[20] Greiwe RM, Saifi C, Ahmad CS (2010). Pediatric sports elbow injuries. Clin Sports Med.

[21] Porcellini G, Rotini R, Kantar SS, Di Giacomo S (2018) Paediatric elbow fractures and rehabilitation. In: The elbow: principles of surgical treatment and rehabilitation. Springer, pp 259–286

[22] Morrey BF, Askew LJ, Chao EY (1981). A biomechanical study of normal functional elbow motion. J Bone Joint Surg.

[23] Nowotny J, Löbstein S, Biewener A, Fitze G, Kasten P (2018). Elbow arthroscopy in children and adolescents: analysis of outcome and complications. Eur J Med Res.

[24] Andelman SM, Walsh AL, Sochol KM, Rubenstein WM, Hausman MR (2018). Arthroscopic elbow contracture release in the pediatric patient. J Pediatr Orthop.

[25] Camp CL, Dines JS, Degen RM, Sinatro AL, Altchek DW (2016). Arthroscopic microfracture for osteochondritis dissecans lesions of the capitellum. Arthrosc Tech.

[26] Bexkens R, van den Ende K, Ogink PT, van Bergen C, van den Bekerom M, Eygendaal D (2017). Clinical outcome after arthroscopic debridement and microfracture for osteochondritis dissecans of the capitellum. Am J Sports Med.

[27] Bojanić I, Smoljanović T, Dokuzović S (2012). Osteochondritis dissecans of the elbow: excellent results in teenage athletes treated by arthroscopic debridement and microfracture. Croat Med J.

[28] Byrd JW, Jones KS (2002). Arthroscopic surgery for isolated capitellar osteochondritis dissecans in adolescent baseball players: minimum three-year follow-up. Am J Sports Med.

[29] Schneider T, Hoffstetter I, Fink B, Jerosch J (1994). Long-term results of elbow arthroscopy in 67 patients. Acta Orthop Belg.

[30] Elfeddali R, Schreuder MH, Eygendaal D (2013). Arthroscopic elbow surgery, is it safe?. J Shoulder Elbow Surg.

[31] Nelson GN, Wu T, Galatz LM, Yamaguchi K, Keener JD (2014). Elbow arthroscopy: early complications and associated risk factors. J Shoulder Elbow Surg.

[32] Marti D, Spross C, Jost B (2013). The first 100 elbow arthroscopies of one surgeon: analysis of complications. J Shoulder Elbow Surg.

[33] Gofton WT, King GJ (2001). Heterotopic ossification following elbow arthroscopy. Arthroscopy.

[34] Intravia J, Acevedo DC, Chung WJ, Mirzayan R (2020). Complications of elbow arthroscopy in a community-based practice. Arthroscopy.

[35] Sodha S, Nagda SH, Sennett BJ (2006). Heterotopic ossification in a throwing athlete after elbow arthroscopy. Arthroscopy.

[36] Keyt LK, Jensen AR, O'Driscoll SW, Sanchez-Sotelo J, Morrey ME, Camp CL (2020). Establishing the learning curve for elbow arthroscopy: surgeon and trainee perspectives on number of cases needed and optimal methods for acquiring skill. J Shoulder Elbow Surg.

[37] Pavone V, Vescio A, Riccioli M, Culmone A, Cosentino P, Caponnetto M, Dimartino S, Testa G (2020). Is supine position superior to prone position in the surgical pinning of supracondylar humerus fracture in children?. J Funct Morphol Kinesiol.

