# Correction to: Pediatric elbow arthroscopy: clinical outcomes and complications after long-term follow-up

**DOI:** 10.1186/s10195-022-00640-z

**Published:** 2022-04-09

**Authors:** Gian Mario Micheloni, Luigi Tarallo, Alberto Negri, Andrea Giorgini, Giovanni Merolla, Giuseppe Porcellini

**Affiliations:** 1 Department of Orthopaedic Surgery, Azienda Ospedaliero Universitaria Di Modena, University of Modena and Reggio Emilia, Modena, Italy; 2 Doctorate School in Clinical and Experimental Medicine, UNIMORE, Modena, Italy

## Correction to: Journal of Orthopaedics and Traumatology (2021) 22:55 https://doi.org/10.1186/s10195-021-00619-2

Following publication of the original article [1], the authors identified an error in the author names. The given name and family name were erroneously transposed.

The incorrect author names: Micheloni Gian Mario, Tarallo Luigi, Negri Alberto, Giorgini Andrea, Merolla Giovanni and Porcellini Giuseppe.

The correct author names: Gian Mario Micheloni, Luigi Tarallo, Alberto Negri, Andrea Giorgini, Giovanni Merolla, Giuseppe Porcellini.

The author group has been updated above and the original article [1] has been corrected.
